# ECOD: new developments in the evolutionary classification of domains

**DOI:** 10.1093/nar/gkw1137

**Published:** 2016-11-28

**Authors:** R. Dustin Schaeffer, Yuxing Liao, Hua Cheng, Nick V. Grishin

**Affiliations:** 1 Department of Biophysics, University of Texas Southwestern Medical Center, Dallas, TX 75390-9050, USA; 2 Howard Hughes Medical Institute, University of Texas Southwestern Medical Center, Dallas, TX 75390-9050, USA

## Abstract

Evolutionary Classification Of protein Domains (ECOD) (http://prodata.swmed.edu/ecod) comprehensively classifies protein with known spatial structures maintained by the Protein Data Bank (PDB) into evolutionary groups of protein domains. ECOD relies on a combination of automatic and manual weekly updates to achieve its high accuracy and coverage with a short update cycle. ECOD classifies the approximately 120 000 depositions of the PDB into more than 500 000 domains in ∼3400 homologous groups. We show the performance of the weekly update pipeline since the release of ECOD, describe improvements to the ECOD website and available search options, and discuss novel structures and homologous groups that have been classified in the recent updates. Finally, we discuss the future directions of ECOD and further improvements planned for the hierarchy and update process.

## INTRODUCTION

Protein three-dimensional structures continue to be determined at an exponential rate, both due to improvements in structure determination techniques and the increase of involved investigators (1–3). Additionally, these structures are increasingly of larger complexes such as ribosomes and viral capsids, mostly due to the rise of cryo-electron microscopy (cryoEM) (4,5). Analysis of domains within newly released protein structures can lead to hypotheses about function and evolutionary origins, but classification of these domains can be time-consuming. Reducing the burden of the domain classification process has typically been achieved by judicious selection of representatives to classify or by implementing automated procedures to supplement, aid and replace elements of the manual curation process (6).

We developed the Evolutionary Classification Of protein Domains (or ECOD) (7) as a hierarchal classification, which emphasizes distantly related homologs that are difficult to detect (H- and X-groups) and takes into account closer sequence-based relationships between protein domains that are placed in families. ECOD exclusively classifies residues in the Protein Data Bank (PDB), i.e. any given residue appears once and only once in the classification. A unique feature of ECOD is that it explicitly classifies domains by topology at a lower level (T-groups), while classifying domains by evolutionary relatedness at a higher level (H-groups) that accounts for significant structural changes in protein evolution. ECOD releases are coupled to PDB releases (with a few weeks delay), such that for every week that there is a PDB release, there is also an ECOD release. We achieve this accelerated update schedule through a combination of automatic and manuals updates: a pipeline, which fully partitions and assigns the majority of proteins in any given week, leaving a fraction that is classified by a manual curator. We have previously discussed challenging examples of manual curation, and shown how strict reliance on structural or sequence similarity scores is insufficient to achieve accurate classification in most difficult cases (8).

### Improved performance of automated ECOD updates

Following the initial release of ECOD (v22), we implemented a weekly update pipeline for ECOD coupled to the release of new structures from the PDB. By quickly and efficiently classifying known structures, and through dedicated manual curation, we are able to classify all depositions in the PDB without overburdening manual curators or relying on solely classifying a reduced set of representative structures. Briefly, the ECOD update pipeline separates a weekly PDB release into a set of individual protein queries based on peptide chains within the PDB depositions (putative fragments or peptides are removed early in the process and either placed into the peptide/fragment categories or reincorporated into ECOD as segments of multi-chain domains). Each member of this set of peptide chains is then individually queried against ECOD reference libraries using a combination of sequence (BLAST, HHsearch) and structural (DALI) aligners (9–12). BLAST alignments to well-covered ECOD reference proteins are used to directly partition query proteins in many (∼90%) cases. Where well-scoring full-protein alignments are not available, individual highly-covered domain hits by BLAST or HHsearch are used to partition unassigned regions of the query.

**Figure 1.**
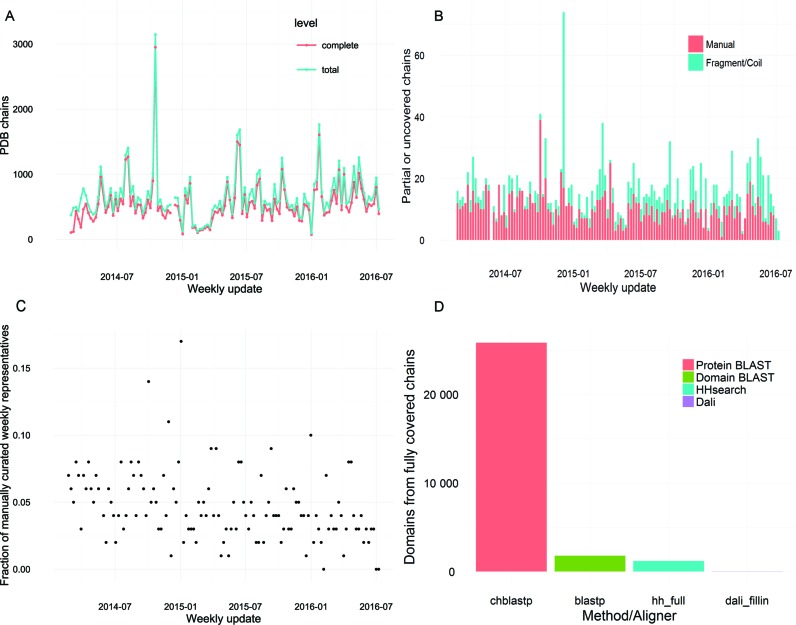
Statistics of Evolutionary Classification Of protein Domains (ECOD) weekly update performance. (**A**) Number of Protein Data Bank (PDB) chains released weekly (total) compared to the number automatically mapped by the ECOD update pipeline (complete). (**B**) Uncovered or partially covered chains are clustered to 95% sequence redundancy and manually curated, chains are either manually assigned to the domain hierarchy or assigned to one of the special architectures (i.e. peptides, coiled coils, synthetics, etc.). (**C**) The fraction of manually curated representatives to total non-redundant chains in a week has been slowly decreasing. (**D**) Methods by which domains from fully assigned chains are determined in ECOD, by full protein–protein BLAST (chblastp), by individual domain–domain BLAST (blastp), by HHsearch domain profile–profile match (hh_full) and by co-determination of neighbor domains by DALI (dali_fillin).

We initiated weekly updates in February 2014 with ECOD version 33. In the following 123 weekly PDB releases, 187 ± 51 structures were classified each week on average (Figure 1A). These classified structures were incorporated into subsequent ECOD releases. Where the automatic update pipeline could generate a putative domain architecture that significantly (>90% and <20 residues uncovered) covered the query chain, those putative domains were assigned to the hierarchy with their hit domains. Where no hits were found, or only a partial domain solution could be resolved, chains were passed along with alignment data to the manual curator (Figure 1B). Curated chains were either assigned to ECOD using a combination of alignment data, functional considerations and/or topological similarities to known domains, or assigned to one of several special architectures, which annotate those residues that are either unclassifiable by our current methodology, or lack sufficient data to be classified in any case (i.e. low resolution structures, peptides, fragments). Total chains partitioned and assigned by manual curation declined over time, and the fraction of manual curation dedicated to assigning unclassified chains to special architectures, rather than the domain hierarchy, increased (Figure 1C). The majority of representative peptide chains (89%) in PDB structures released between 2014–2016 were classified by protein–protein BLAST. A total of 6% of classified domains are classified by domain-domain BLAST, and 4% by profile–profile detection using HHsearch. Only 20 domains were classified by DALI co-domain detection (0.06%). Of the chains classified by protein–protein BLAST, 78% were single-domain (Figure 1D). The efficacy of the automated toolchain and the low proportion of previously uncharacterized proteins in weekly PDB releases allow for the timely of manual curation of the remaining chains.

Those chains that cannot be partitioned into domains and assigned automatically are manually curated (8). The result of manual curation is either to partition a peptide chain into domains and to assign those domains to H-groups within the ECOD hierarchy, or to assign a chain, in part or wholly, to one of the ECOD special architectures. In either case, the manual assignment of these domains or special architecture regions helps to automate subsequent assignments. Over time, our manual curation workload has remained steady in the face of the gradually increasing rate of structure releases, and the ratio of domains to non-domain (e.g. coiled-coil, peptide, synthetic) regions has decreased. We attribute these shifts to the change in focus from small, structurally novel, globular domains, to larger complexes wherein associated domains may contain more extended regions in the deposited coordinates increasing numbers of extended structures in recent PDB releases likely result from their physical ordering by protein–protein interactions within large complexes, or from a shift from X-ray crystallography refinement techniques, where disordered regions are more likely to be excluded from modeled regions, to microscopy refinement techniques, where large disordered regions are more likely to be included in the deposited structural coordinates.

### Addition of ECOD representative sets

ECOD is highly redundant with respect to sequence and structural similarity. Since the release of ECOD we have departed from our F-group model of Pfam HMM-based families mixed with HHsearch-based single-linkage clusters. Now we use families described by Hidden Markov Models (HMMs), some of our own creation. With the increased week-to-week stability and performance that this change provided, we were able to recalculate ECOD representative sets on a weekly basis and provide them as a standard distribution. Domain clusters are generated using BLASTCLUST at 99%, 70% and 40% sequence filtering over 90% alignment coverage of both the query and the reference sequences. Because H-groups can contain multiple T- and F-groups, filtering only occurs between members of the same F-group. This rule ensures that each F-group is represented at least once, although representatives may be sequence-similar above the level of filtering to members of other F-groups. Representatives are selected for each version from clusters preferring (i) existing F40 representatives (i.e. from previous versions), (ii) manually curated domains, (iii) provisional manual domains, (iv) domains from structures determined by X-ray crystallography, (v) domains from structures with higher X-ray crystal resolution, (vi) and domains from more recently released structures. These representative sets are made available, along with their PDB structure sets, at our webpage (http://prodata.swmed.edu/complete/distributions).

### New ECOD web search methods

**Figure 2.**
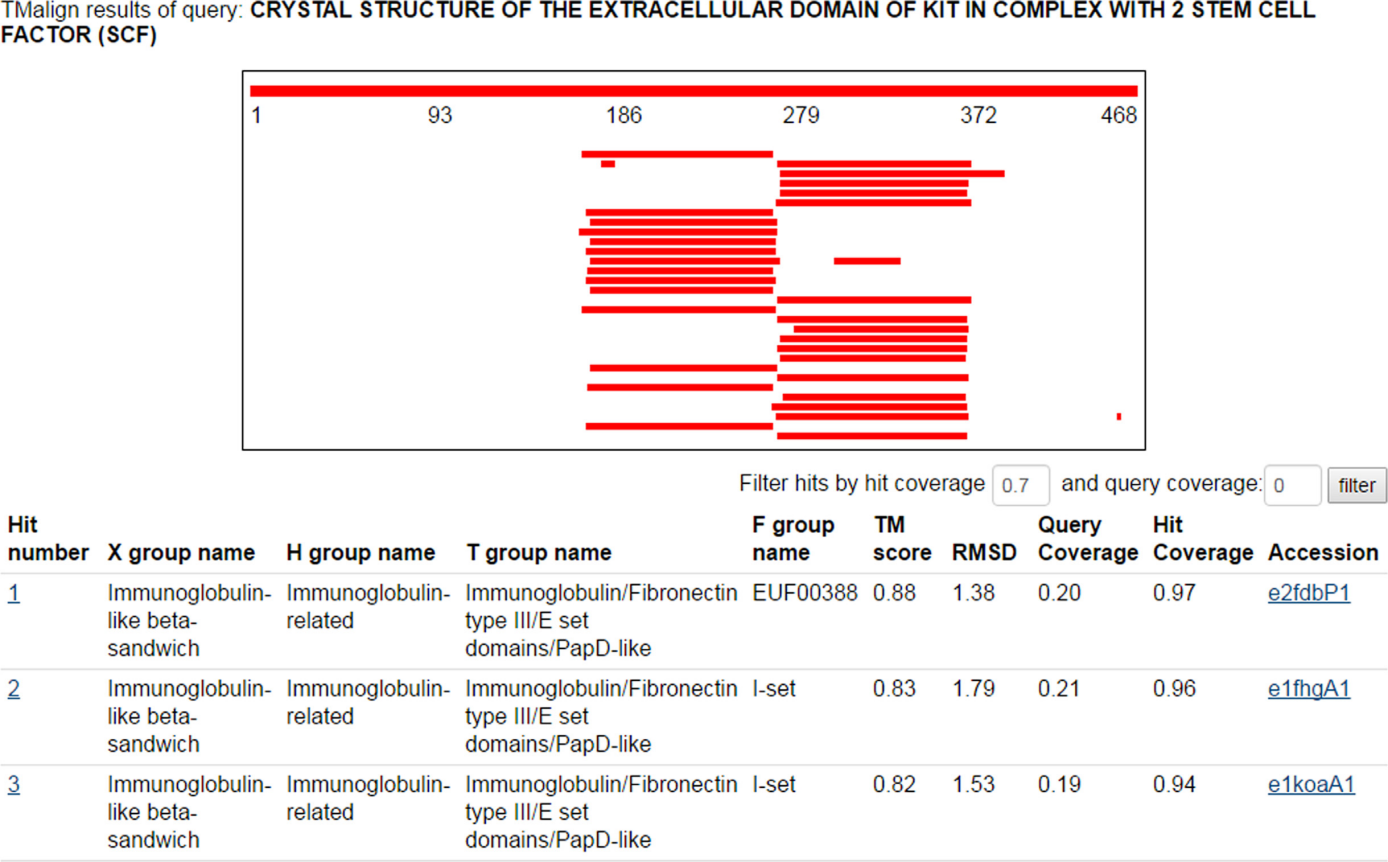
Screenshot of ECOD webpage Tm-align search results for KIT ectodomain (2e9w, A), showing Ig domain hits in two regions. Lower section shows ECOD hierarchy data for hits, along with TM-align hit statistics.

We have expanded the search utilities available for searching ECOD from the web. It is now possible to search ECOD using a user-input query structure with TM-align (13) against the F40 representative domain set of the current release. The top hits are first summarized in a graph in which individual bar represents aligned region and is colored based on TM-score (Figure 2). The list of results can be filtered both by query coverage and hit coverage to exclude partial structure matches (e.g. which happens frequently for helical domains). For each hit, a JSmol (14) viewer displaying the hit structure with aligned region colored in rainbow and a downloadable Pymol session file of the superposition are available.

ECOD can also be searched by sequence using COMPADRE, our recently released distant homology search program (15). COMPADRE utilizes knowledge of homology network in the classification database and can boost scores significantly based on combined information from all homologs of one particular hit. Now ECOD has been added to the COMPADRE search database that is updated periodically. We provide an option to search ECOD database using COMPADRE method and default parameters on the ECOD website.

When browsing the database in tree view, users can now select domains of interest to perform a MUSTANG multiple structure alignment (16). Output alignments and RMSD matrices constructed by the MUSTANG program are displayed in the results along with a links to a Pymol session of the structural superposition. We found it useful for exploring the homology relationships in the classification, such as studying shared features among families in the same H-group and also comparing different H-groups.

### Classification of ECOD sequence families

ECOD provides evolutionary classification of domains into homologous (H-groups) and potentially homologous groups (X-groups). In addition, we partition groups of homologous domains into sequence families of closer relatives, frequently with similar functions. Because family classification in itself is to a large degree reliant on manual identification of functional motifs and interaction partners, we rely on the well-established classification provided by the Pfam sequence database to the extent possible (17). Putative ECOD domains are first assigned to H/T-groups (few H-groups have more than a single T-group). Manual domains are assigned directly to T-groups by curators, whereas automatic domains are assigned using the T-group of the ECOD hit-domain (i.e. the reference domain with the highest sequence and/or similarity score). Final classification of both manually curated and automatically partitioned domains is made by detection of similarity to HMMs generated from sequence family multiple alignments curated in the Pfam sequence database (17). Pfam v27 contained 14 836 individual sequence families. Of these, 6126 classify, wholly or in part, one or more sequence families in ECOD. A total of 8710 Pfam v27.0 families remain unlinked to any ECOD F-group. These unlinked Pfam families are principally families that were either further split in ECOD into several domains, proteins of unknown function, viral proteins or coiled-coils.

Some domains are not yet classified in Pfam. In order to provide more consistent classification within ECOD, we endeavor to provide provisional sequence families where more comprehensive sequence curation is not yet available. Those domains that have no good Pfam sequence models are clustered for non-redundancy, then used as queries for JACKHMMER (http://hmmer.org) searches against the UniRef90 non-redundant protein database (18,19). The resultant alignments are used to build provisional sequence families for inclusion into ECOD using HMMBUILD. These ECOD Unclassified Families (or EUFs) are considered to be provisional, and are more likely to be subject to future reclassification. ECOD v149 referenced 6560 EUFs that were used to generate 41% of ECOD F-groups, however, F40 representative domains from F-groups mapped solely by EUFs constitute only 36% of the ECOD representative set. Novel sequence families tend to contain fewer domains and be from more recently deposited structures. Validating these EUFs is an ongoing project within ECOD.

A further complication of F-group/sequence family classification is the need to ensure that new sequence families have their representative domains chosen. Our ability to efficiently manipulate the database during updates of ECOD is partially due to strict manual representative/non-representative mappings between domains: when a manual representative is updated or removed, it is trivial to map those changes onto associated non-representatives. We require that manually curated representatives and their automatic non-representatives belong to the same F-group within ECOD. However, the automatic assignment process can assign based on distant homologous relationships that span sequence families. Consequently, sequence families can be formed that have no manual representative. In this case, domains have manual representatives that exist in other sequence families. In order to maintain at least one representative per sequence family, a single automatic domain is selected from these manually unrepresented families and designated as a ‘provisional manual representative.’ These provisional domains are indicated by an asterisk on the website. In ECOD v149, 25% of F-groups and 12% of F40 domains are represented by only provisional manual representatives, respectively.

### Classification of new homologous groups

F-groups may be created by the automatic update pipeline, but groups on all other hierarchal levels (X, H and T) must be created through manual curation and populated with at least one representative. Although not all curation results in the creation of new groups, on average 2.5 new X-groups (no clear homology to any known domain) and 1.9 new H-groups (some inconclusive evidence of homology to known domains) were created and populated in each weekly update. Proteins assigned to these new groups were generally partitioned as single-domain (60%), with a smaller fraction partitioned into more than one domain (40%). Domains in newly created X-groups are not homologous to any other ECOD domain; however, they may gain new homologs in subsequent versions. Of the 273 X-groups and 220 H-groups defined by manual curation during the weekly updates, 75% of both have received additional domains subsequent to their inception. Many of these additions are redundant, considering only the addition of new F40 domains, only 14% and 12% have received new additions, respectively. These novel PDB chains are generally not classified by other structural domain classifications. Only proteins observed in 19 and 27 of the new ECOD X- and H-groups are observed in SCOPe v2.06. Similarly, only proteins within 31 of the new ECOD X-groups and 34 of the new ECOD H-groups are observed in CATH v4.1.

**Figure 3.**
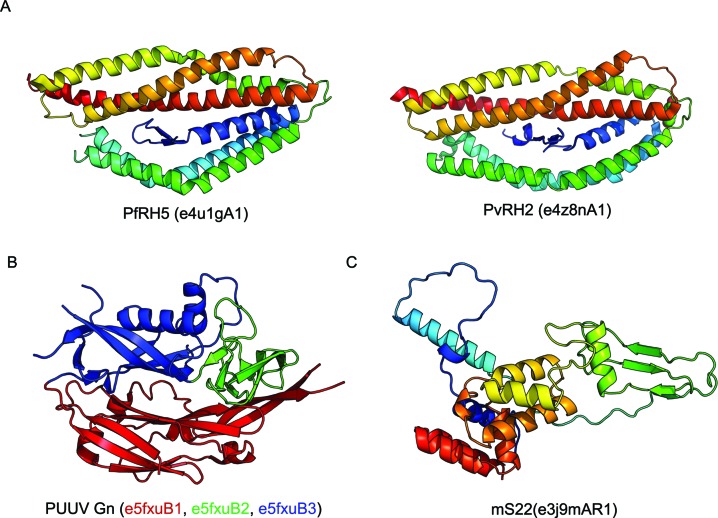
Examples of domains in new X- and H-groups assigned by manual curation in ECOD. (**A**) Novel reticulocyte-binding protein homologue (RH) domain PfRH5 (e4u1gA1) did not pass automatic classification and was used as the seed for a new X-group. A subsequent domain from *Plasmodium vivax* was added to this new X-group automatically. (**B**) Pumala hantavirus (PUUV) Gn polyprotein was divided into three domains, 2 of which were novel, by manual curation. (**C**) A novel mitoribosomal subunit, ms22, contains biomedically relevant mutation sites and was recently classified along with other novel mitoribosomal structures.

One motivation for the accelerated update schedule of ECOD is to collect recent structures of biomedical interest as quickly as possible and make their relationships available to the community. The reticulocyte-binding protein homologue (RH) protein family is necessary for invasion of host erthyrocytes in *Plasmodium falciparium*. The structure of RH5 from *Plasmodium* was determined and found to have a novel alpha-helical fold that may form an obligate multimer (20,21). The novelty of the fold was confirmed by ECOD curators, the structure was determined to be single-domain and the RH5 domains (e4u1gA1, e4watA1) were placed into an newly created X-group (Figure 3A). A subsequent structure of a RH protein (4z8n) from *P. vivax* was added to ECOD by the pipeline automatically as a provisional manual domain (22).

New groups in ECOD can contain domains from viral proteins. The fast evolution of viral proteins can be responsible for significant structural variation and elaboration. The initial 2.3 A X-ray structure of the hantavirus glycoprotein Gn (5fxu) ectodomain (23) was manually partitioned into three domains: a beta-sandwich into a new F-group within existing ECOD Ig domain H-groups, and two novel domains which were placed into two new X-groups(Figure 3B). The novel domains consisted of (i) an SH3-like domain (e5fxuB2) that contained (ii) an inserted beta barrel-like domain (e5fxuB3). No additional domains have been subsequently classified with a shared X- or H-group to the Gn hantaviral domains. By seeding ECOD with new X-groups for structurally novel domains, we can provide for automatic classification of their homologs in the future.

The mamallian mitoribosome, although descendant from alpha-proteobacteria, contains many unique structural features (24). The structure of both the 39S and 28S subunits, as well as the full 55S mitoribosome structure, has recently been determined by single-particle cryoEM (25–28). Classifying structures determined by cryoEM is technically challenging in several aspects. CryoEM structures may contain higher proportions of disordered regions in their constituent proteins, or regions that are only ordered by contact with other complex members. The collected high-resolution structures of the human ribosome yielded several structurally unique members. mS22, mS23, mS26, mS31, mS33, ms38 and ms38 each nucleated a new H-group. In only two cases, ms23 and ms34, sufficient similarity was detected to add these H-groups to existing X-groups, RuvA-like and SH3-like, respectively. In ms22, there are clinically significant mutations: R170H and L125P, the structural effects of which are still being studied (29,30) (Figure 3C). We expect that the number of clinically significant protein complexes will continue to grow as a fraction of the ECOD curation load in the future.

## CONCLUSION

The mission of ECOD is to provide timely and consistent domain classification of proteins released by the PDB. We maintain a unique database uniquely focused on the full classification of the available structures, and by full coverage of the residues in those structures. Here, we have presented some of the improvements to website search and the result of more than 18 months of consistent weekly updates. In the future we intend to focus on making quality-control data available through the same portal, formalizing our description of sequence F-groups, and describing the inter-domain interfaces present in the protein complexes in which ECOD domains participate. We anticipate that the types of structures classified by ECOD will continue to shift toward large complexes determined by microscopy methods, and that the fraction of single-domain X-ray crystal structures will continue to decrease. This shift will require a continued focus on the automated treatment of these structures, and the curation tools used to classify them manually.
